# Application of the Superelastic NiTi Spring in Ankle Foot Orthosis (AFO) to Create Normal Ankle Joint Behavior

**DOI:** 10.3390/bioengineering4040095

**Published:** 2017-12-07

**Authors:** Amirhesam Amerinatanzi, Hashem Zamanian, Narges Shayesteh Moghaddam, Ahmadreza Jahadakbar, Mohammad Elahinia

**Affiliations:** Dynamics and Smart Systems Laboratory, Mechanical, Industrial and Manufacturing Engineering Department, The University of Toledo, Toledo, OH 43606, USA; Amirhesam.Amerinatanzi@rockets.utoledo.edu (A.A.); Hashem.Zamanian@rockets.utoledo.edu (H.Z.); Narges.ShayestehMoghaddam@rockets.utoledo.edu (N.S.M.); ahmadreza.Jahadakbar@rockets.utoledo.edu (A.J.)

**Keywords:** superelastic NiTi spring, Ankle Foot Orthosis (AFO), normal ankle joint behavior

## Abstract

Hinge-based Ankle Foot Orthosis (HAFO) is one of the most common non-surgical solutions for the foot drop. In conventional HAFOs, the ankle joint is almost locked, and plantar flexion is restricted due to the high stiffness of the hinge mechanism. This often leads to a rigid walking gate cycle, poor muscle activity, and muscle atrophy. Since the ankle torque-angle loop has a non-linear profile, the use of a superelastic NiTi spring within the hinge, due to its nonlinear behavior, could recreate a close-to-normal stiffness of the normal ankle joint, which, in turn, could create a more natural walk. The focus of this study is to evaluate the performance of a superelastic NiTi spring versus a conventional Stainless Steel spring in a hinge mechanism of a custom-fit HAFO. To this aim, a custom-fit HAFO was fabricated via the fast casting technique. Then, motion analysis was performed for two healthy subjects (Case I and Case II): (i) subjects with bare foot; (ii) subjects wearing a conventional HAFO with no spring; (iii) subjects wearing a conventional Stainless Steel-based HAFO; and (iv) subjects wearing a NiTi spring-based HAFO. The data related to the ankle angle and the amount of moment applied to the ankle during walking were recorded using Cortex software and used for the evaluations. Finally, Finite Element Analysis (FEA) was performed to evaluate the safety of the designed HAFO. The NiTi spring offers a higher range of motion (7.9 versus 4.14 degree) and an increased level of moment (0.55 versus 0.36 N·m/kg). Furthermore, a NiTi spring offers an ankle torque-angle loop closer to that of the healthy subjects.

## 1. Introduction

Walking is the most basic form of human motion. Millions of people in the world are unable to walk normally because of gait disabilities [1]. Foot drop gait or steppage gait is a kind of gait in which the dropping of the forefoot happens due to the weakness of the ankle and foot dorsiflexors. There are two common complications caused by drop foot. First, the patient cannot control the falling of their foot after a heel strike. As a result, the foot slaps the ground on every step. The second complication is the inability of the patients to clear their toe during the swing phase. This causes the patients to drag their toe on the ground throughout the swing phase [2,3,4].

Up to now, different routes have been investigated to design better assistive devices to enable patients to walk closer to normal [5,6,7,8,9]. Ankle Foot Orthosis (AFO) is the most common non-surgical solution for the foot drop [9,10]. In general, there are three types of ankle foot orthotic (AFO) devices: passive devices, semi-active devices, and active devices. Passive AFOs provide stability during walking and standing by maintaining the joints in a fixed position. Locking the ankle joint in this type of AFOs causes excessive knee flexion moment during loading response, which leads to rigid walking gait cycle, lower muscle activity, and muscle atrophy. Posterior leaf spring AFO or flexible AFO is a semi-rigid plastic AFO that assists push-off during pre-swing and prevents drop-foot [11,12,13]. In posterior leaf spring AFOs, the ankle joint has limited dorsi and plantar flexion. Active AFOs can interact with the walking environment and act accordingly. Active AFOs allow, or have the potential to allow, sagittal plane motion at the ankle. These types of AFOs are composed of a variety of hinges, flexion stops, and stiffness control elements like spring, oil damper, one-way friction clutch, electronic control systems, and actuators [14,15,16]. 

Blaya et al. [17,18,19] developed a powered ankle-foot orthosis based on Series Elastic Actuators composed of a DC motor, mechanical links, and torsional springs. The basic idea of the Active Ankle Foot Orthosis (AAFO) is to change the orthosis impedance (stiffness) actively, which eliminates the slap foot. As a result, the AAFO minimizes the kinematic walking difference compared to normal people. Ferris et al. designed other active AFOs that are pneumatically powered. This AAFO is actuated by McKibben Muscles, which are pneumatic actuators. One pneumatic actuator creates plantar flexion torque, and, on the other hand, the second actuator creates dorsiflexion torque. This study has shown promising results in gait rehabilitation, human motor adaptation, and muscle activations [20,21,22,23].

Mataee et al. [24] presented two innovative, adaptive solutions for the AFOs based on the mechanical and structural stiffness control of the shape memory alloys. These concepts address gait abnormality in drop foot patients for various walking conditions such as different walking speeds. In their first design, a superelastic rod provides variable torsional stiffness that is adjusted by a controlled axial load. In the second design, the active length of the superelastic hinge is adjusted to control the bending stiffness of the element. Bdahane et al. [25,26,27] presented another novel active AFO by using superelastic wires to help patients who have foot drop. Their device has fourteen plastic pulleys, which were fixed on the AFO by using screws and spacers, making a parallel combination of eight wires. The results of this study indicate that superelastic wires provide controlled plantar flexion in the stance phase and active dorsiflexion in the swing phase due to stiffness variation of the SMA wires. These kinds of active AFOs need complex control systems, and their use is limited to rehabilitation devices in lab environments. 

In recent years, different types of Hinge-based Ankle Foot Orthoses (HAFOs) have been modified. This type of active AFOs not only has a simple design but also has efficient functionality that creates normal behavior in the ankle joint. Conventional hinge joints such as the Klenzak ankle joint with the Stainless Steel spring are used to control the motion of the ankle in the sagittal plane. Yamamota et al. [28,29] developed a hinged ankle foot orthosis with a dorsiflexion assist. In this study, a conventional AFO with a Klenzak ankle joint has been modified to prevent drop foot during walking. Their modified design is called DACS AFO and has a spring in the posterior part of the tibial upright of the AFO, which generates plantar flexion resistance moment during heel strike and prevents foot-slap. This design can provide 2–17 N·m dorsiflexion assisting moments per 10 degrees of plantar flexion. The stiffness of the DACS AFO can be changed by inserting different Stainless Steel springs in the hinge zone. Using a Stainless Steel spring in these designs creates a limitation in the normal movement of the ankle [14,30,31]. This abnormality causes excessive knee flexion moment during loading response, which leads to rigid walking gait cycle, lower muscle activity, and muscle atrophy. Recently, shape memory alloy-based AFOs have also been introduced and studied [32,33,34,35]. The proposed designs were able to provide the required motion and assist patients; however, it is often hard to control the SMA component during cooling. 

The focus of this study is to evaluate the performance of a superelastic NiTi spring versus the conventional Stainless Steel spring, within the hinge mechanism of a custom-fit HAFO. Since superplastic NiTi spring has nonlinear behavior in elongation and compression, it has been hypothesized that the resulting hinge could bring the stiffness of the ankle closer to that of the healthy ankle joint, which could lead to more natural walking in drop foot patients. To this aim, a Stainless Steel hinge, as well as several Stainless Steel and NiTi springs, were created with different diameters. Subsequently, a custom-fit HAFO was fabricated via the fast casting technique. The results for the ankle moment and the ankle angle indicate that using the superelatic NiTi spring offers ankle moment and ankle angle profiles closer to the healthy subject.

## 2. Methods

### 2.1. Custom-Fit Ankle Foot Orthosis (AFO)

**Figure 1:**
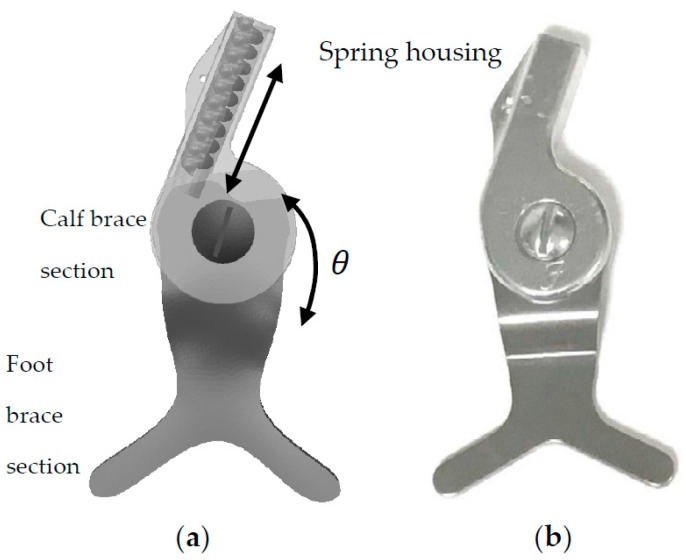
(**a**) The Computer-aided design (CAD) and (**b**) the fabricated Stainless Steel hinge. The spring can be compressed inside the spring housing due to the relative rotation between the calf and foot brace sections. The existence of the spring smoothens the walking of the patient wearing Hinge-based Ankle Foot Orthosis (HAFO).

To produce a hinge-based ankle foot orthosis, a conventional Stainless Steel hinge (Dorsiflexion Assist Ankle Joint Kit, Fillauer Companies, Chattanooga, TN, USA) was retrofitted to include appropriate room for mounting springs (Figure 1). 

**Figure 2:**
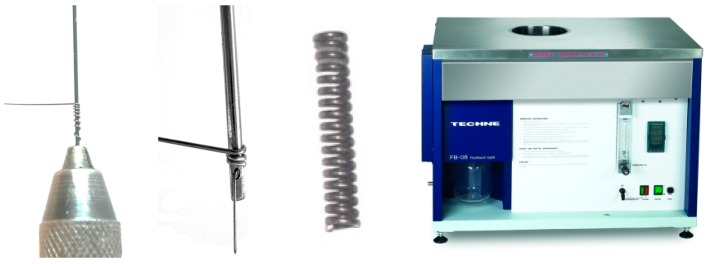
The production process of the NiTi spring. In this process, a NiTi wire is first wrapped around the NiTi wire, and then the shape setting procedure is performed to create the spring.

To create the conventional hinge, several Stainless Steel springs with the inner diameter of 2.5 mm and wire diameter of 0.68 mm were provided. For the proposed NiTi-based hinge, the NiTi springs were created from NiTi wires with the diameters of 0.68 and 1.07 mm (Fort Wayne Metals, Fort Wayne, IN, USA). As it is seen in Figure 2, the procedure of spring fabrication included: (i) wrapping the wire around a mandrel with the fold thicknesses of 2.5 mm (similar to the inner diameter of conventional Stainless Steel springs); and (ii) performing heat treatment process for shape setting the wire. According to Liu et al. [36], to stabilize the spring, the appropriate shape setting process was performed in a fluidized baths furnace (FB08, Techne Inc., Staffordshire, UK) at the temperature of 500 °C for 10 min followed by water quenching (M_f_ = −88 °C, M_s_ = −65 °C, A_s_ = −23 °C, and A_f_ = −8 °C). Subsequently, the springs were embedded within the hinge, one after another.

**Figure 3:**
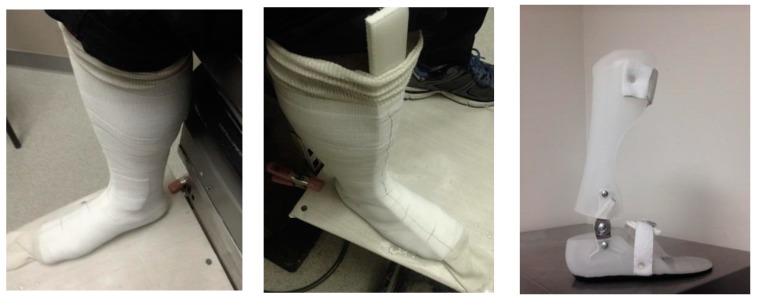
Patient-specific Ankle Foot Orthosis (AFO) casting.

Finally, as shown in Figure 3, a custom-fit AFO was created using the fast casting technique. The AFO was composed of two separate sections, which were connected to each other via a spring-based hinge.

### 2.2. Motion Analysis and Data Analysis

**Figure 4:**
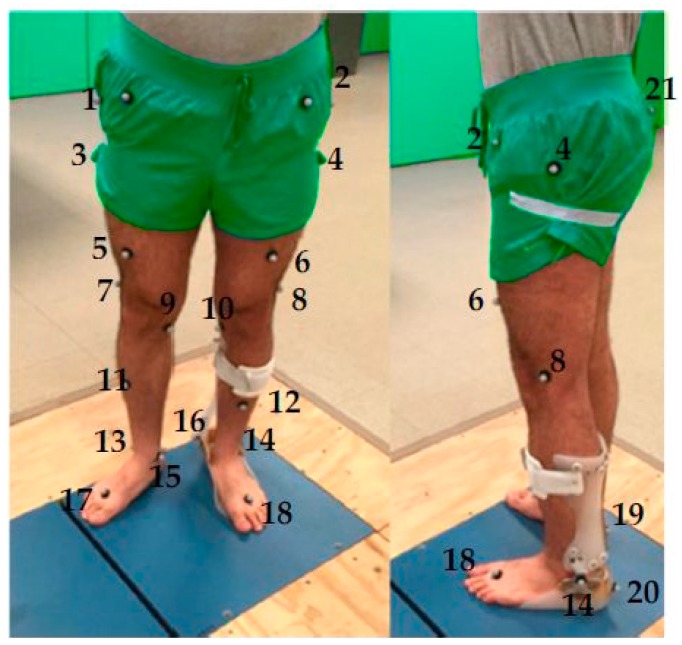
Reflective markers on the lower half of the subject wearing HAFO. (Markers 1 and 2: Ilium Crest Tubercle, markers 3 and 4: Femur greater Trochanter, markers 5 and 6: right and left thigh, markers 7 and 8: Femur Lateral Epicondyle, markers 9 and 10: Femur Medial Epicondyle, markers 11 and 12: right and left shank, markers 13 and 14: Fibula Apex of Lateral Malleolus, markers 15 and 16: Tibia Apex of Medial Malleolus, markers 17 and 18: Head of 2nd Metatarsus, markers 19 and 20: Posterior Surface of Calcaneus, and marker 21: Sacrum (mid-point between mid-point between right ilium posterior superior (RIPS) and left ilium posterior superior (LIPS)).

To evaluate the performance of the HAFOs, four different conditions were evaluated for two healthy subjects (Case I: 31-year old, male, 75 kg, height 175 cm and Case II: 30-year old, male, 72 kg, height 176 cm): (i) subjects with bare foot; (ii) subjects wearing conventional HAFO with no spring; (iii) subjects wearing conventional Stainless Steel-based HAFO; and (iv) subjects wearing NiTi spring-based HAFO. For each condition, 4 trials have been done. Twenty-one reflective markers were attached to predefined locations on the subjects based on Helen Hayes marker set, as shown in Figure 4 [24,25]. 

**Figure 5:**
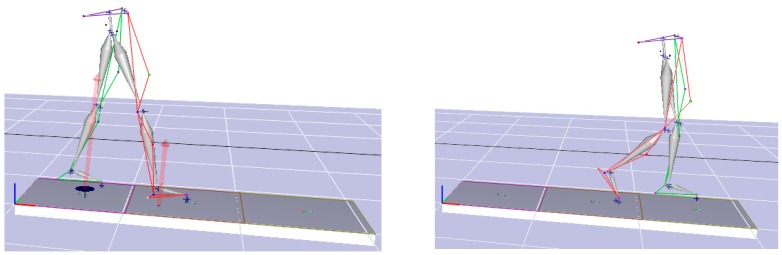
Cortex software environment during motion analysis test.

Ten cameras Raptor-E digital real-time analysis system (Motion Analysis, Inc., Irvine, CA, USA) with a rate of 120 Hz and three Optima Force plates (Advanced Mechanical Technology, Inc., Watertown, MA, USA) with a rate of 720 Hz were used to collect the kinetic and kinematic data during each test. The data of stiffness profiles (normalized moment per subject weight via ankle angle) were reported after filtration (a 6 Hz low pass Butterworth filter) in Cortex^®^ software (Toronto, ON, Canada), as shown in Figure 5.

### 2.3. Finite Element (FE) Modeling

**Figure 6:**
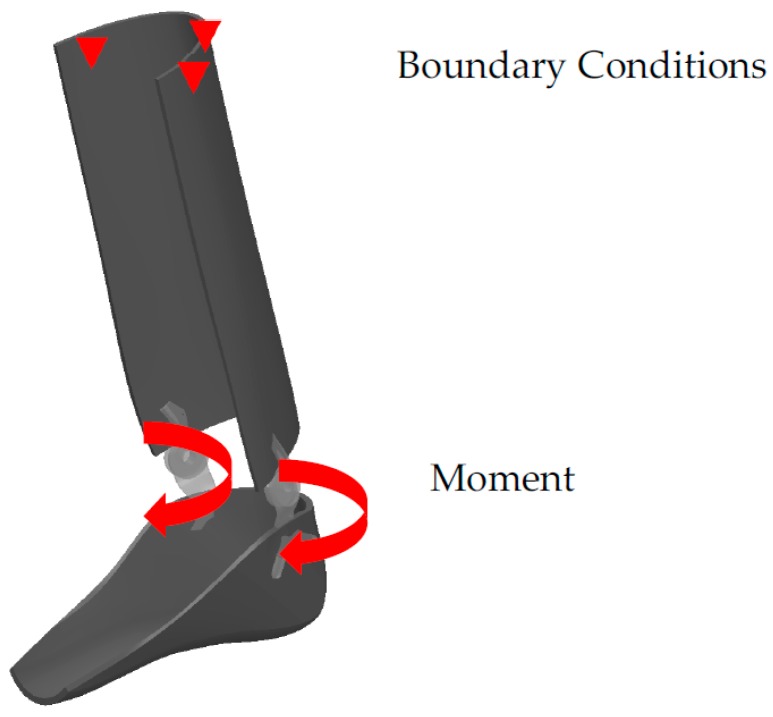
The 3D assembly of the AFO components and the applied moment and boundary conditions.

To evaluate the reliability of the HAFOs, FE analysis was performed on the designed AFO using ABAQUS (CAE v6.11, Dassault Systems, Providence, RI, USA). The required dimensions (foot length, height, etc.) are those of the healthy subject. Then, all components (hinges, AFO brace, and springs) were virtually designed in Solidworks (Dassault Systèmes, Waltham, MA, USA) based on real dimension of the healthy subject. Figure 6 shows the 3D assembly of the AFO components.

All the components were meshed in Hypermesh (Hyperworks, Detroit, MI, USA) with 10-node tetrahedral elements (C3D10). To assure that the model outputs are independent of mesh size, all 3D structures were meshed with different mesh density. The peak stress under a defined constant tension load was recorded for all different mesh densities. The optimized number of elements is reported in Table 1 based on the maximum 5% convergence tolerance in model output for each component [37].

The interactions between the hinge/AFO and hinge/springs were considered as “tie”, and a “self-contact interaction” was considered for the spring. The material properties of hinge, AFO, and springs are summarized in Table 2 [11,25,28,38]. As shown in Figure 6, in the simulation, to obtain the distribution of von Mises’ stress in the maximum plantar flexion, an external moment of 0.62 N·m/kg was applied to the center of rotation of the hinge. The value of the moment has been obtained from ankle torque-angle graph. The maximum stress in the NiTi spring at maximum plantar flexion has been used for calculation of the safety factor. Moreover, superior part of the calf braced has been fixed. 

Then, two models were run in a dynamic mode: (i) considering the properties of Stainless Steel spring; and (ii) considering the material properties of superelastic NiTi by using a User Material (UMAT) [39].

**Figure 7:**
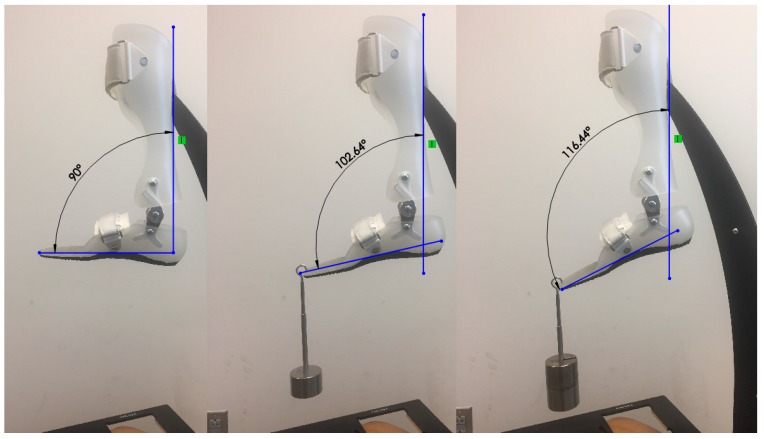
Experimental setup to define the changes in the motion of the HAFO by applying force.

For the validation of the model, an experimental setup shown in Figure 7 is considered. In this experiment, the range of motion of the HAFO was calculated via changing the external loading. Then, the resultant experiments were compared to those of finite element analysis.

Safety factor is calculated by dividing maximum allowable stress by equivalent stress. Ultimate tensile strength for NiTi is around 900 MPa. By extracting von misses stress, distribution for AFO at maximum planter flexion safety factor has been calculated.

## 3. Results and Discussion

### 3.1. Ankle Torque-Angle Profile

**Figure 8:**
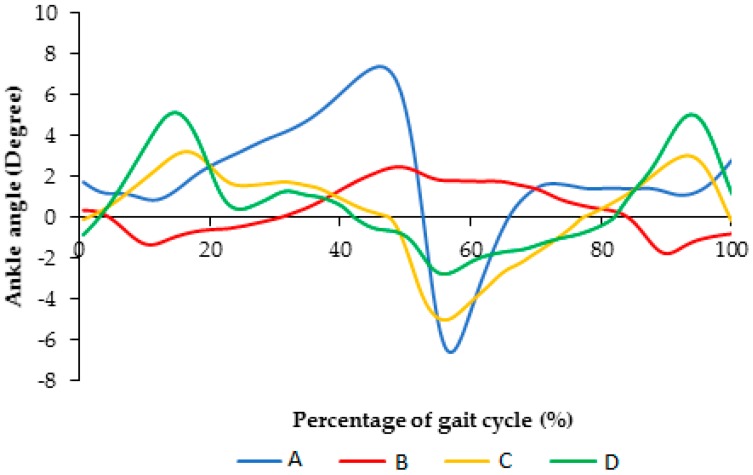
Ankle angle versus percentage of gait cycle during normal walking (A); using AFO with Stainless Steel spring (B); and using AFO with superelastic NiTi spring (C and D). (Case II).

Normal walking has two portions: stance and swing phases. During the stance phase, the foot contacts the ground. This phase starts with heel strike and continues until toe off. At the moment of toe off, the swing phase starts and lasts while the foot is in the air. The next cycle initiates with another heel strike when the foot strikes the ground again. The resultant mean ankle angles versus the percentage of gait cycle are presented in Figure 8. As it shows, use of Stainless Steel springs limited ankle flexion to between 1.5° to 2.6° in dorsi and plantar flexion, respectively. However, superelastic NiTi caused a higher range of flexion, more than 4° plantar-flexion, and about 4° dorsiflexion. 

**Figure 9:**
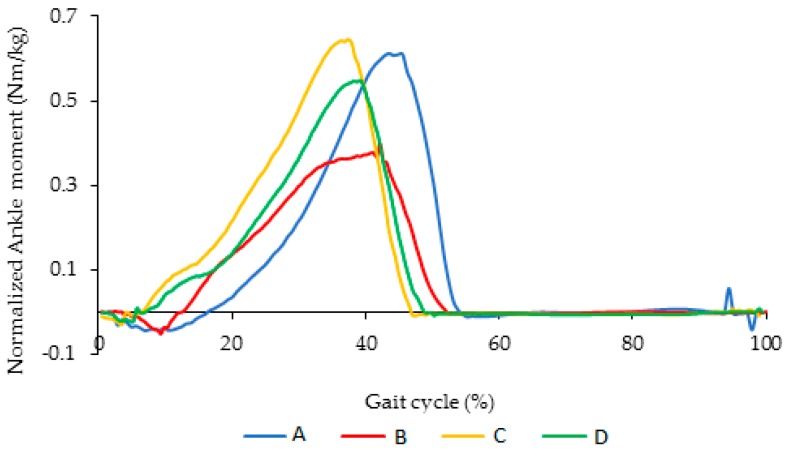
Normalized ankle moment versus percentage of gait cycle during normal walking (A); AFO with Stainless Steel spring (B); and using AFO with superelastic NiTi spring (C and D). (Case II).

Figure 9 illustrates the normalized ankle moment for normal walking with respect to the percentage of gait cycle for a healthy subject (Case II), the subject using AFO with Stainless Steel spring, and the subject using AFO with superelastic NiTi springs (type I and type II). As it can be seen in Figure 9, the maximum ankle moment for a healthy subject is 0.62 (N·m/kg), but the maximum ankle moment for AFO with conventional Stainless Steel spring is 0.4 (N·m/kg). So, the difference would be 0.22 (N·m/kg), which could be 155.4 (N·m) for a subject with 72 kg weight. Reduction in ankle moment due to reduction of range of motion is the reason for abnormal and unstable walking. 

**Figure 10:**
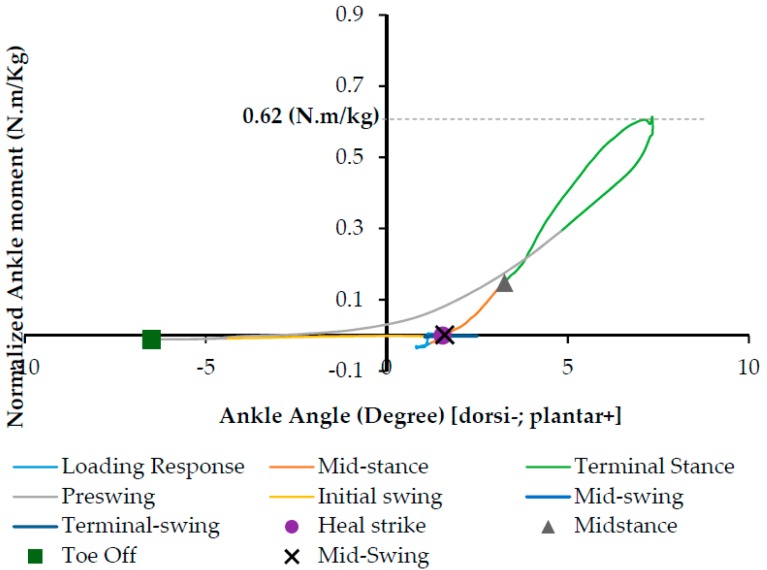
Ankle torque-angle loop for a healthy subject during normal walking.

Ankle torque-angle loop profile can be considered as normalized ankle moment in respect to ankle rotation (plantar/dorsiflexion). Ankle torque-angle loop is the resistance of the ankle joint in response to an applied ankle rotation during the gait cycle [13]. Different studies have been done on healthy male and female subjects to extract ankle torque-angle loops during the normal walking [14,17,20,21]. Ankle torque-angle loop demonstrates the generation and absorption of mechanical energy at the ankle during the gait cycle. By combining the data of the ankle moment and angle, ankle torque-angle loop for a subject during normal walking is presented in Figure 10. As shown in Figure 10, the gait phases of a person walking on the ground have been shown in ankle torque-angle loop. 

### 3.2. Evaluation of the Proposed AFO vs. Conventional AFO

**Figure 11:**
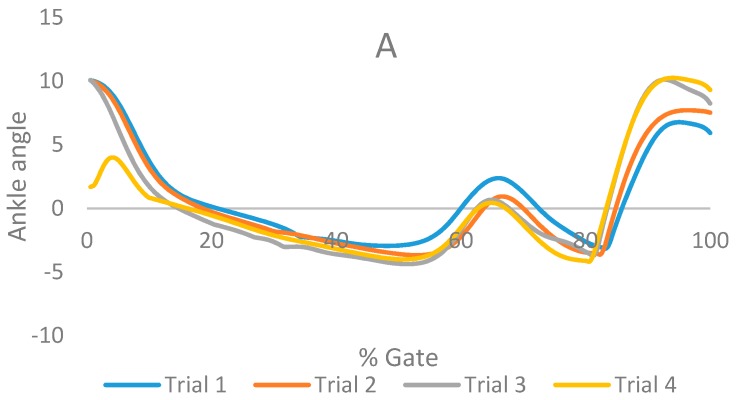

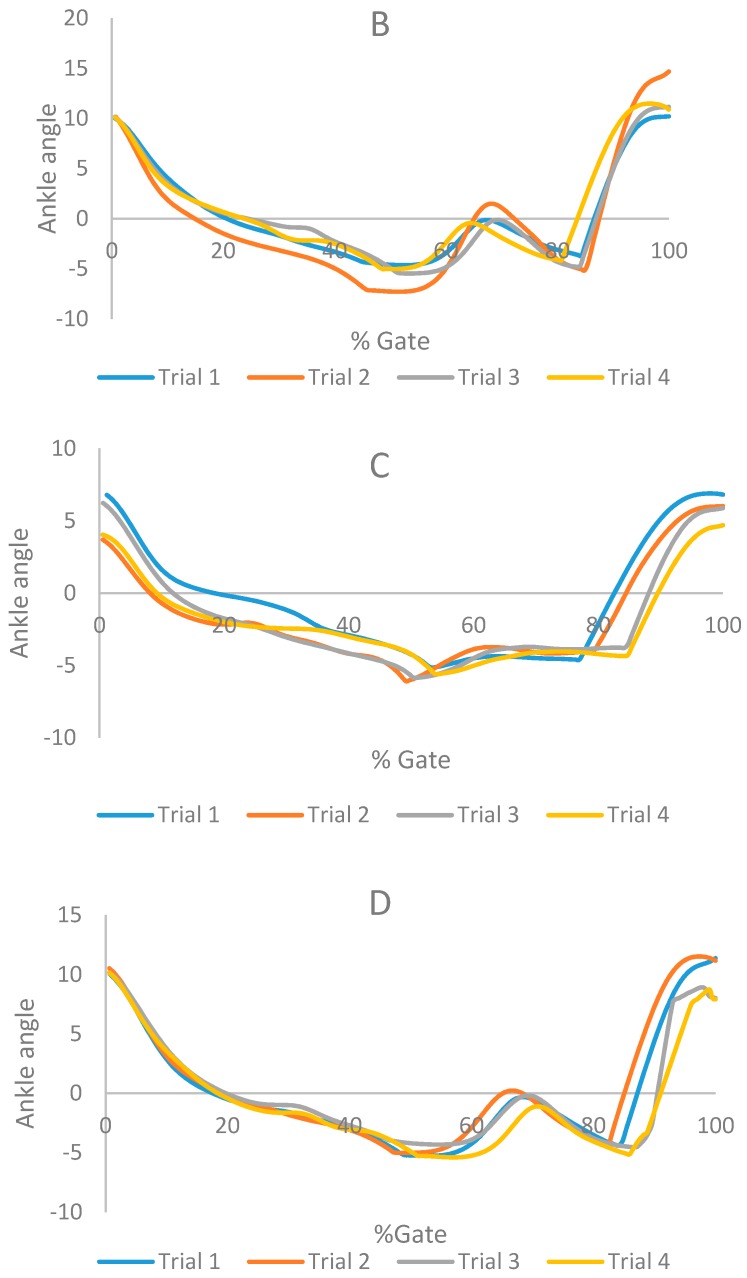
Ankle angle for each trial for different conditions including (**A**) subject with bare foot; (**B**) subject wearing conventional HAFO with no spring (**C**) subject wearing conventional Stainless Steel-based HAFO; and (**D**) subject wearing NiTi spring based HAFO. (Case I).

For the healthy subject (case I), Figure 11 illustrates ankle angle for each trial for different conditions including (A) subject with bare foot, (B) subject wearing conventional HAFO with no spring (C), subject wearing conventional Stainless Steel-based HAFO, and (D) subject wearing NiTi spring-based HAFO. 

**Figure 12:**
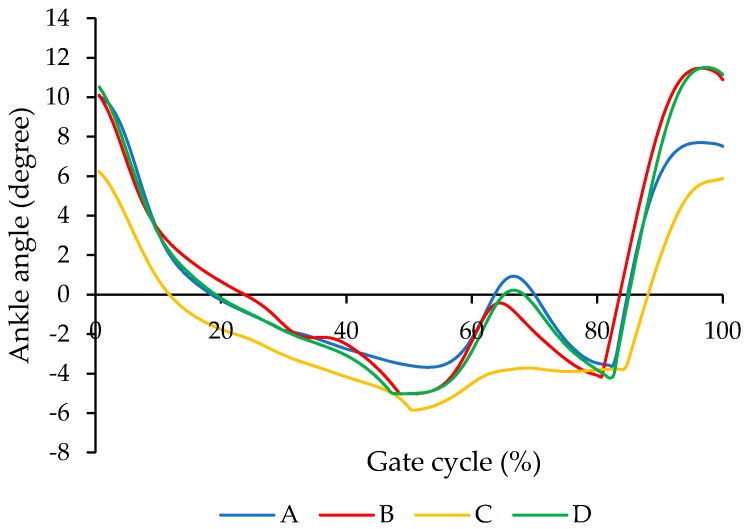
Comparison of the ankle angle for each trial for different conditions including (A) subject with bare foot; (B) subject wearing conventional HAFO with no spring; (C) subject wearing conventional Stainless Steel-based HAFO; and (D) subject wearing NiTi spring.

Figure 12 illustrates the comparison of the ankle angle for different conditions including (A) subject with bare foot, (B) subject wearing conventional HAFO with no spring (C), subject wearing conventional Stainless Steel-based HAFO, and (D) subject wearing NiTi spring-based HAFO. As shown here, ankle angle for the condition that we have for the NiTi spring in the AFO is closer to the barefoot condition. Using the bare foot condition as the baseline, root mean square error (RMSE) was calculated as 2.95 and 1.16 for conditions C and D, respectively. Having more of a range of motion in the AFO and having the ankle angle close to the bare foot condition altogether promises a more normal walking. 

**Figure 13:**
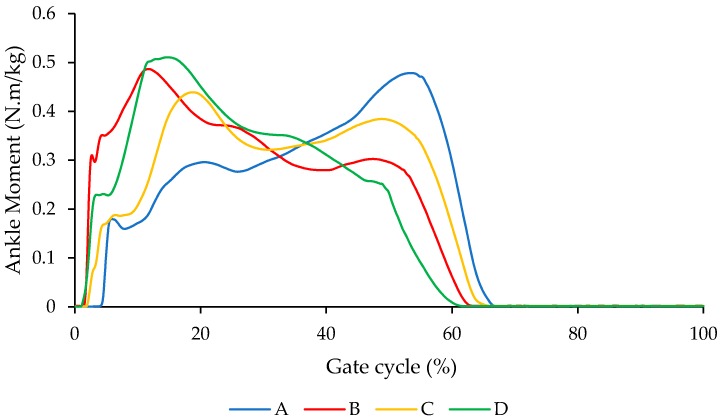
Comparison of the ankle moment for different conditions including (A) subject with bare foot; (B) subject wearing conventional HAFO with no spring; (C) subject wearing conventional Stainless Steel-based HAFO; and (D) subject wearing NiTi spring based HAFO. (Case I).

Figure 13 illustrates the comparison of the ankle moment for different conditions A, B, C, and D. As shown in Figure 13, ankle moment for the condition that we have for the NiTi spring in the AFO is closer to barefoot condition.

**Figure 14:**
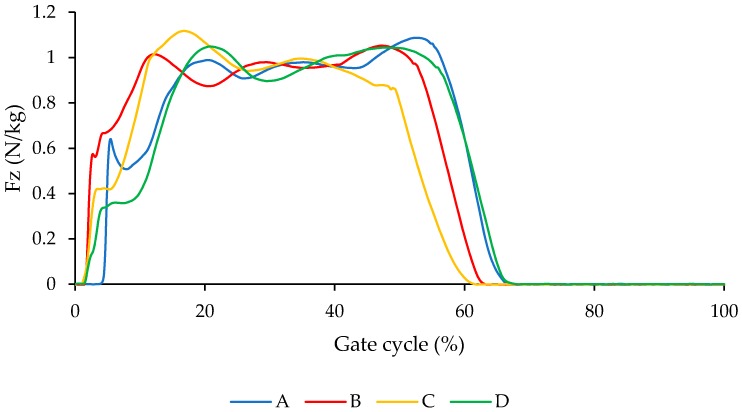
Fz (vertical component of ground reaction force (GRF)) for conditions (A) subject with bare foot; (B) subject wearing conventional HAFO with no spring; (C) subject wearing conventional Stainless Steel-based HAFO; and (D) subject wearing NiTi spring-based HAFO. (Case I).

Figure 14 illustrates the comparison of Fz (vertical component of ground reaction force GRF) for different conditions A, B, C, and D. As shown in Figure 14, Fz for the condition that we have for the NiTi spring in the AFO is closer to the barefoot condition.

**Figure 15:**
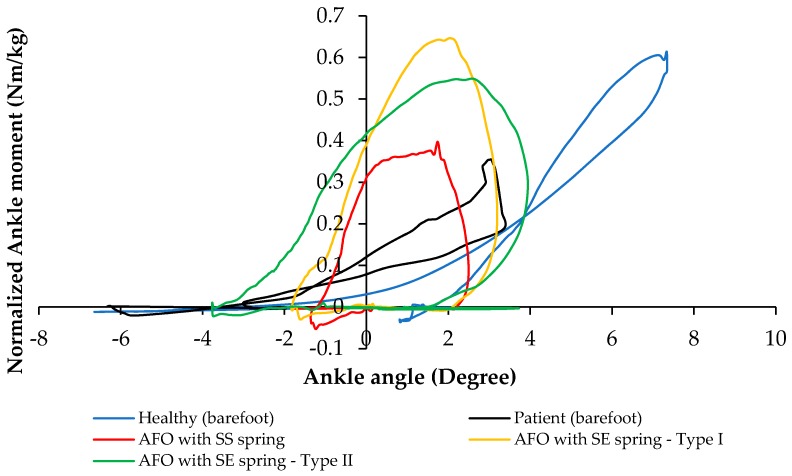
Ankle torque-angle loop during normal walking (blue), AFO with Stainless Steel spring (red), AFO with superelastic NiTi spring and diameter of 0.68 mm (yellow), and AFO with superelastic NiTi spring and diameter of 1.07 mm (green) (Case II) [42].

To evaluate the functionality of the NiTi springs in the AFO, ankle torque-angle loops of the subject wearing different types of AFOs have been plotted in Figure 15. The blue graph shows ankle torque-angle loop of a healthy subject. The resultant torque-angle loop of the subject using the AFO with Stainless Steel spring (red), superelastic NiTi spring with the diameter of 0.68 mm (yellow), and superelastic NiTi spring with the diameter of 1.07 mm (green) are presented here. As can be seen in Figure 15, in normal walking of a healthy subject, ankle joint has maximum 7 degrees of plantar flexion and 7 degrees of dorsiflexion. Conventional AFO with Stainless steel spring creates an abnormal walking, because it does not allow for enough range of motion. This can be attributed to the high stiffness and also to the linear behavior of the conventional Stainless Steel spring. Another disadvantage of using the conventional stiffness spring is that it will not lead to a desired level of ankle joint moment. As seen in Figure 15, the ankle moment for a healthy subject has a maximum value of 0.62 (N·m/kg), but ankle moment for AFO with conventional Stainless Steel spring creates about 0.4 (N·m/kg) ankle moment at its maximum value. Using superelastic NiTi springs creates a better range of motion in the ankle joint. As shown in Figure 15, Superelastic NiTi spring with the diameter of 0.68 mm (yellow) creates 5 degrees of dorsiflexion and 3 degrees of plantar flexion, which is closer to the range of motion of the healthy subject. In this case, ankle moment has a maximum value of 0.65 (N·m/kg), which is close to the maximum value of ankle moment for the healthy subject 0.62 (N·m/kg). Superelastic NiTi spring with diameter of 1.07 mm (green) creates 3 degrees of dorsiflexion and 5 degrees of plantar flexion, which is also closer to the range of motion of the healthy subject. In this case, ankle moment has a maximum value of 0.54 (N·m/kg), which is relatively close to the maximum value of ankle moment for the healthy subject 0.62 (N·m/kg). As seen in Figure 15, ankle torque-angle loop for a healthy subject has a nonlinear profile with maximum ankle moment of 0.62 (N·m/kg). Considering ankle torque-angle loop for a healthy subject as the base line, in this study, it has been tried to find a spring which can create an ankle torque-angle loop close to the healthy subject. As seen in Figure 15, ankle torque-angle loop for the condition that we have in the NiTi spring in the AFO is closer to the healthy subject. Having more of a range of motion in the AFO and having the ankle torque-angle loop close to the healthy subject altogether promise more normal walking. Having nonlinear ankle torque-angle loop when we have NiTi spring in AFO can be attributed to nonlinear behavior of NiTi in the elongation and compression. 

**Figure 16:**
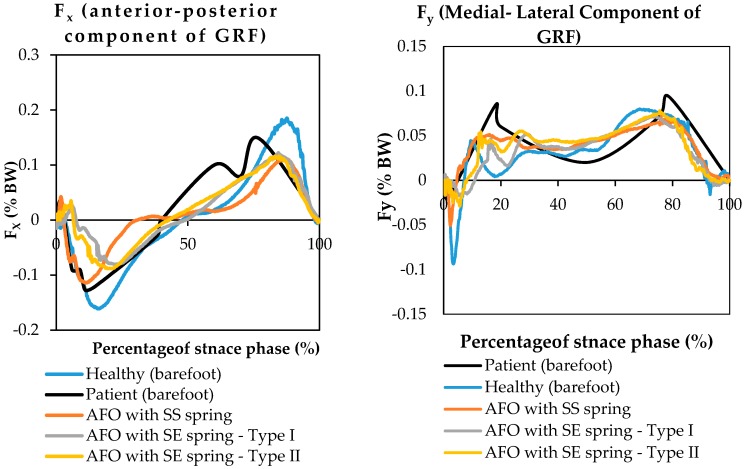
GRF during normal walking using AFO with Stainless Steel spring, AFO with superelastic NiTi spring (0.68 mm), and AFO with superelastic NiTi spring (1.07 mm) (*x*, *y*, and *z* axis are presenting Anterior-Posterior, Medial-Lateral, and vertical components of ground reaction force) (Case II) [42].

Ground reaction forces have been illustrated with respect to frame number during normal walking using AFO with Stainless Steel spring, AFO with superelastic NiTi spring (0.68 mm), and AFO with superelastic NiTi spring (1.07 mm) Figure 16. 

**Figure 17:**
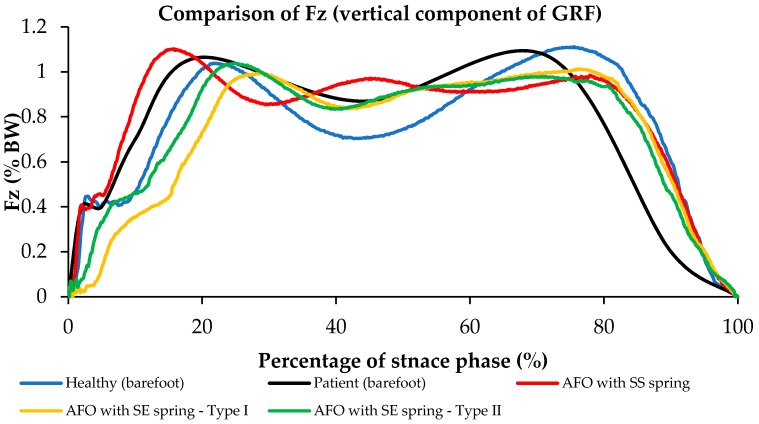
Comparison of Fz (vertical component of GRF) during normal walking using AFO with Stainless Steel spring, AFO with superelastic NiTi spring (0.68 mm), and AFO with superelastic NiTi spring (1.07 mm) (Case II) [42].

Comparison of F_z_ (vertical component of GRF) during normal walking using AFO with Stainless Steel spring, AFO with superelastic NiTi spring (0.68 mm), and AFO with superelastic NiTi spring (1.07 mm) has been shown in Figure 17. The vertical component of GRF (F_z_) during normal walking has two peaks. The first peak is due to weight acceptance during stance. After the first peak, during the midstance, due to knee flexion, the GRF drops below the body weight level. During the push of phase the second peak will occur, and after this the seconds peak F_z_ will decrease to zero, when toe off will occur and foot will be disconnected from the force plate. The vertical component of GRF during walking using AFO with Stainless Steel spring has three peaks, which is abnormal and is not close to normal walking. The vertical component of GRF during walking using AFO with superelastic NiTi spring has two peaks, which is much closer to normal walking.

**Figure 18:**
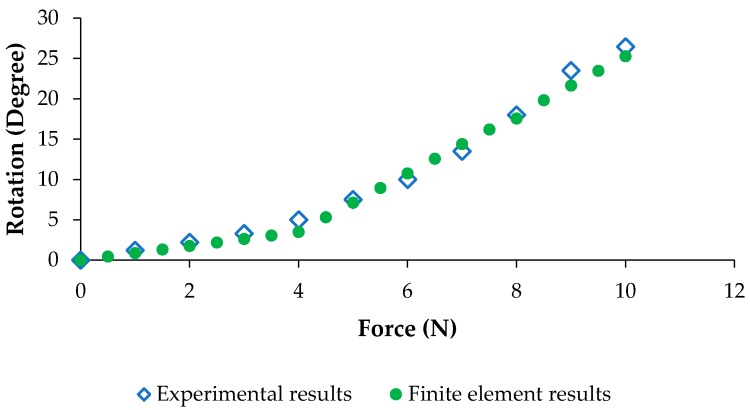
Model validation: a comparison between experimental data with FE model data.

The results of the experimental setup are compared with the simulation to evaluate the validity of the model. As it is shown in Figure 18, the simulation results are in agreement with the experimental ones. 

**Figure 19:**
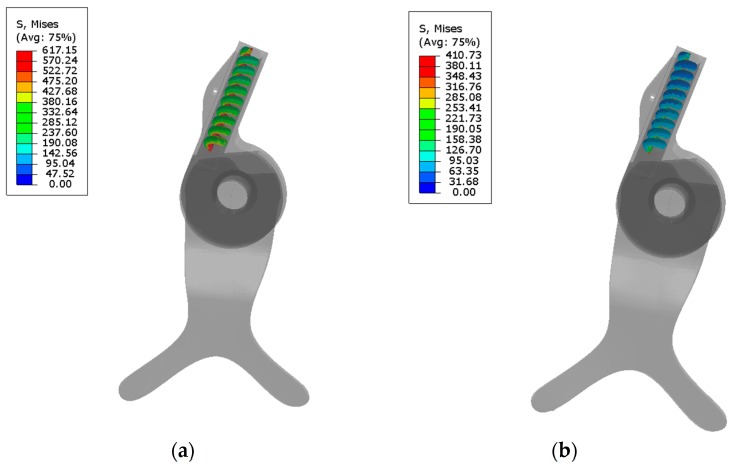
Von Mises stress distribution at the maximum plantar flexion: (**a**) Stainless Steel, (**b**) NiTi spring. (Unit = MPa).

The distribution of von Mises’ stress in the maximum plantar flexion for the stainless steel springs (about 50% of gait cycle) and NiTi springs (about 15% of gait cycle) are shown in Figure 19. FEM analysis showed that during terminal stance, before heel off, during which maximum plantar flexion occurs (5 degrees plantar flexion), the maximum stress on the superelastic NiTi spring would be 410 MPa. As it was discussed, safety factor is calculated by dividing maximum allowable stress by equivalent stress. Ultimate tensile strength for NiTi is around 900 MPa. Maximum stress at NiTi spring for AFO at maximum planter flexion is 410 MPa. So, safety factor would be 2.19.

**Figure 20:**
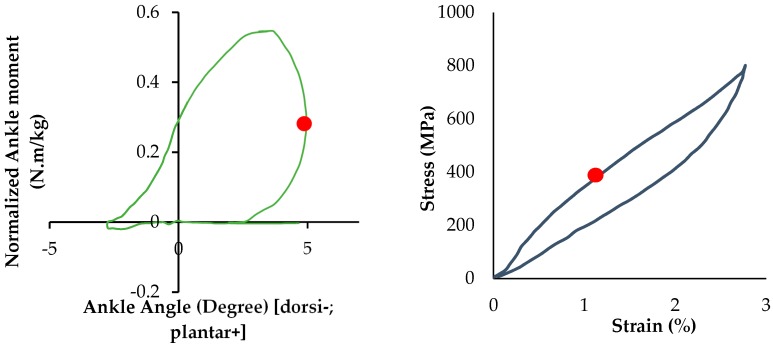
Corresponding stress at the maximum plantar flexion in NiTi stress).

As it shown in Figure 20, the NiTi springs reach to the upper plateau zone when the springs reach to the maximum plantar flexion (at about 15% of gait cycle). In fact, due to large deformation of the NiTi springs, a greater range of plantar flexion occurred compared to conventional that in Stainless Steel springs.

## 4. Summary and Conclusions

In this study, for the first time, the use of the superelastic NiTi spring in the HAFO was evaluated. To this aim, several motion analyses were conducted on (i) the healthy subject; (ii) the subject wearing the conventional Stainless Steel-based HAFO; and (iii) the subject wearing the NiTi-based HAFOs. A comparison study has been conducted to find a desire wire size that will lead to a more normal walking pattern. FE analysis was also performed to evaluate the safety of the NiTi-based HAFO. 

The proposed study has several limitations that need to be improved in the future work. First, the current device was designed for someone weighing 72 kg. It might not be able to provide enough support for those who exceed this weight. The HAFO should be custom-made for different users to better meet specific requirements. Second, only one subject was involved in the motion tests. The sample size is far from adequate and provides limited information in assessing the HAFO. Future studies are planned to improve the design and increase the number of subjects (of both healthy people and patients with drop foot) and to collect more data to evaluate the functionality and reliability of the device on various users. Third, in this study the moments were reported based on GRF which does not give enough muscle activity evaluation data. More studies need to be conducted using electromyogram (EMG) measurements on subjects.

The main conclusions are as follows:

A normal ankle will have about 14 degrees range of motion. Therefore, it is important to design an AFO which could create a higher range of motion in comparison with the current AFOs in order to create more normal walking for the patients.

This study showed that the use of superelastic NiTi springs with a diameter of 1.07 mm can allow a higher plantar flexion angle (about 2.5°) compared to the use of stainless steel springs. In the other words, the superelastic NiTi springs are not only capable of allowing plantar flexion closer to the normal walking situation, but also they can lead to more dorsiflexion of the foot compared to conventional designs.

It can be concluded that during walking using AFO with Stainless Steel, there would be less ankle flexion after the first peak, so GRF does not drop as normal walking. In addition, during walking using AFO with superelastic NiTi spring, there would be more ankle flexion after the first peak, so GRF drops as normal walking.

For the proposed HAFO compared to the conventional HAFO, we observed a higher range of motion (7.9 versus 4.14 degree) and an increased level of ankle joint moment (0.55 N·m/kg versus 0.36 N·m/kg).

FEM analysis showed that during the terminal stance before heel off, during which maximum plantar flexion occurs (5 degrees plantar flexion), the corresponding maximum stress on the superelastic NiTi spring would be 410 MPa. During these periods, the spring is in the upper plateau phase. So in these cases, due to large deformation of the NiTi springs, a higher range of plantar flexion is allowed compared to that in the conventional stainless steel springs.

## Figures and Tables

**Table 1:** The number of elements for the Finite Element Analysis model components.

Model Component	Element	
	Type of Element	Number of Element
AFO (Bottom)	3D, Solid, Tetrahedral, Deformable	26,437
AFO (Top)	3D, Solid, Tetrahedral, Deformable	65,179
Spring (4 components)	3D, Solid, Tetrahedral, Deformable	26,058
Hinge (Top)	3D, Solid, Tetrahedral, Deformable	13,090
Hinge (Bottom)	3D, Solid, Tetrahedral, Deformable	21,037

**Table 2:** Material properties of the model components [40,41].

Component	Hinge	AFO	SE Spring	SS Spring
Material	Stainless steel	Plastic	Superelastic NiTi	Stainless steel
Young’s Modulus (GPa)	220	20	30 */40 **	210
Poisson’s Ratio	0.33	0.27	0.33	0.33
Martensitic Start (M_s_)	-	-	−65	-
Austenitic Start (A_s_)	-	-	−23	-
Martensitic Finish (M_f_)	-	-	−88	-
Austenitic Finish (A_f_)	-	-	−8	-

* E_M_: Martensitic modulus of elasticity, ** E_A_: Austenitic modulus of elasticity.
